# Determining Factors Affecting Nurses’ Acceptance of a Care Plan System Using a Modified Technology Acceptance Model 3: Structural Equation Model With Cross-Sectional Data

**DOI:** 10.2196/15686

**Published:** 2020-05-05

**Authors:** Kuei-Fang Ho, Pi-Chen Chang, Maria Dyah Kurniasari, Sri Susanty, Min-Huey Chung

**Affiliations:** 1 Department of Nursing Ching Kuo Institute of Management and Health Keelung Taiwan; 2 School of Nursing College of Nursing Taipei Medical University Taipei Taiwan; 3 Department of Nursing Faculty of Medicine and Health Science Universitas Kristen Satya Wacana Salatiga, Central Java Indonesia; 4 Department of Nursing Faculty of Medicine University of Halu Oleo Kendari, Southeast Sulawesi Indonesia; 5 Department of Nursing Shuang-Ho Hospital Taipei Medical University New Taipei City Taiwan

**Keywords:** care plan system, technology acceptance model 3, behavioral intention

## Abstract

**Background:**

Health information technology is used in nursing practice worldwide, and holistic patient care planning can serve as a guide for nursing practice to ensure quality in patient-centered care. However, few studies have thoroughly analyzed users’ acceptance of care plan systems to establish individual plans.

**Objective:**

Based on the technology acceptance model 3 (TAM3), a user technology acceptance model was established to explore what determines the acceptance of care plan systems by users in clinical settings.

**Methods:**

Cross-sectional quantitative data were obtained from 222 nurses at eight hospitals affiliated with public organizations in Taiwan. Using the modified TAM3, the collected data were employed to analyze the determinants of user acceptance of a care plan system through structural equation modeling (SEM). We also employed moderated multiple regression analysis and partial least squares–SEM to test the moderating effects.

**Results:**

We verified all significant effects from the use of a care plan system among bivariate patterns in the modified TAM3, except for moderating effects. Our results revealed that the determinants of perceived usefulness and perceived ease of use significantly influenced perceived usefulness and perceived ease of use, respectively. The results also indicated that nurses’ perceptions of subjective norm (path coefficient=.25, *P*<.001), perceived ease of use (path coefficient=.32, *P*<.001), and perceived usefulness (path coefficient=.31, *P*<.001) had significantly positive effects on their behavioral intention to use the care plan system, accounting for 69% of the total explained variance.

**Conclusions:**

By exploring nurses’ acceptance of a care plan system, this study revealed relationships among the variables in TAM3. Our results confirm that the modified TAM3 is an innovative assessment instrument that can help managers understand nurses’ acceptance of health information technology in nursing practice to enhance the adoption of health information technology.

## Introduction

Nurses’ ability to develop detailed care plans considerably influences the quality of patient care [1]. Care plans are essential tools for promoting holistic care and are used to guide the practice of, communication about, and recording of the provided care in routine care settings [2,3]. Suitable individual care plans have been associated with correct medical observations and appropriate nursing diagnoses [4-6]. Therefore, it is reasonable to infer that such care plans lead to the appropriate implementation of care, accurate judgments of achieved patient goals, and clinically effective nursing interventions. In nursing environments, informatics has been used to improve data management and promote care planning [7]. With the help of information technology, a care plan system was developed to facilitate the planning, organization, coordination, and recording of the nursing process.

Several models have been proposed to examine the factors affecting individual reactions to information technology. For example, the user acceptance of technology model is the most popular model used to evaluate information systems [8]. The technology acceptance model (TAM) identifies why individuals adopt new technologies in various domains and is a popular topic of research in the information systems field. The original TAM contains two belief constructs, namely perceived usefulness (PU) and perceived ease of use (PEOU), which have been defined by Venkatesh and Davis [9] and Venkatesh [10], respectively (see Table 1). These constructs determine an individual’s behavioral intention (BI) toward using information technology; PU has a stronger and more direct impact than does PEOU [9-11].

Venkatesh and Bala [11] developed a theoretical framework for TAM-related research by synthesizing prior research conducted on the TAM. This theoretical framework involves the social influence, systemic characteristics of determinants, individual differences, and facilitating conditions related to PU and PEOU. Social influence encompasses the social processes and mechanisms that shape individuals’ perceptions of various aspects of a technology. Systemic characteristics refer to the identity of a system and can help individuals perceive the ease of use and usefulness of said system. Individual differences are personal characteristics or demographics that influence PEOU and PU. Finally, facilitating conditions refer to organizational infrastructure and support, which promote the adoption of a technology in a given context. Venkatesh and Bala [11] combined a theoretical model of the determinants of PEOU and PU with the original TAM and called this extended model TAM3. This model has since proven to be reliable and highly accurate for predicting and explaining user acceptance of various forms of information technology.

Theoretical processes such as social influence and cognitive instruments explain the relationship between PU and its determinants (ie, subjective norm [SN], image [IMG], job relevance [REL], and result demonstrability [RES]). SN and IMG are categorized as social influence processes, whereas REL and RES are system characteristics that reflect the effects of cognitive instrumental processes. Furthermore, according to the theoretical framework of TAM3, individual differences and facilitating conditions explain the determinants of PEOU through the anchoring and adjustment of human decision making. Anchoring involves four constructs, namely perception of external control (PEC), computer self-efficacy (CSE), computer anxiety (CANX), and computer playfulness (PLAY). These constructs reflect how individuals anchor the PEOU of a target system to their beliefs. The adjustment of perceived enjoyment (ENJ) and objective usability modifies individuals’ PEOU of a target system. Objective usability is determined through the comparison of the amount of time spent by an expert with that spent by a novice to perform a task using the system [10]. The specific definitions of the determinants of PU and PEOU are provided in Table 1.

The variables of the original TAM have the power to predict nurses’ technological acceptance of and intention to use information technology [12,13]. One study employed the original TAM to explore nurses’ acceptance of a nursing information system for care planning. The researchers reported that PEOU and PU significantly influenced nurses’ acceptance levels [14]. Zhang et al [15] conducted a study on the determinants of PU in the context of mobile homecare nursing to better understand the acceptance of a technology.

After reviewing the literature on user acceptance of a nursing information system, we noted that most studies were based on the original TAM only or theories regarding the determinants of PU. In addition to studying the relationships of REL and RES with PU, Zhang et al [15] observed that SN and IMG within an organization were significant antecedents of PU and that PU was the most influential factor in the adoption of mobile information technology by homecare nurses. In other words, to date, few studies have examined the determinants of PEOU or developed a combined model of the determinants of PEOU and PU in the context of nursing information system use. The care plan system in this study was developed by the North American Nursing Diagnosis Association on the basis of their classification system and was validated by our previous research [16]. This paper presents an empirical study on this care plan system that incorporated the modified TAM3 to explore the acceptance mechanism of a care plan system. The objectives of this study were to (1) identify the determinants of nurses’ acceptance of a care plan system and (2) determine the influence of bivariate patterns in the modified TAM3 on the use of a care plan system.

**Table 1:** Definitions of constructs in the modified technology acceptance model 3.

Construct				Definition
**Perceived usefulness**				The degree to which an individual believes that using a technology will enhance his or her job performance [9]
	**Social influence**			
		Subjective norm	An individual’s perception of whether the people who are important to them think that they should use the target system [9]	
		Image	The degree to which an individual perceives that using a technology will enhance their image or status in their social circle [9,17]	
	**Cognitive instruments**			
		Job relevance	One’s perception of a technology as facilitative to their job [9]	
		Result demonstrability	An individual’s perception of the tangible (observable and communicable) results from using the target system [9,17]	
**Perceived ease of use**				The degree to which an individual believes that using a technology will be free of effort [10]
	**Anchoring**			
		Perception of external control	Individuals’ perceptions regarding the availability of organizational responses to facilitate the use of a target system [11]	
		Computer self-efficacy	Individuals’ beliefs regarding their abilities to use information technology [11]	
		Computer anxiety	An individual’s degree of fear or apprehension when they use or consider using a target system [10,18]	
		Computer playfulness	The degree of perceived spontaneity in an individual’s interaction with a technology [10]	
	**Adjustment**			
		Perceived enjoyment	The performance-related consequences of using a target system and the degree to which using said system is perceived to be enjoyable [10]	
**Moderator**				
		Output quality	The strength of individuals’ beliefs regarding how well a system enables the performance of a task with respect to said individuals’ job goals [10]	
		Voluntariness	The rating range of voluntary use of a target system [10]	

## Methods

### Theoretical Framework of the Technology Acceptance Model 3

**Figure 1:**
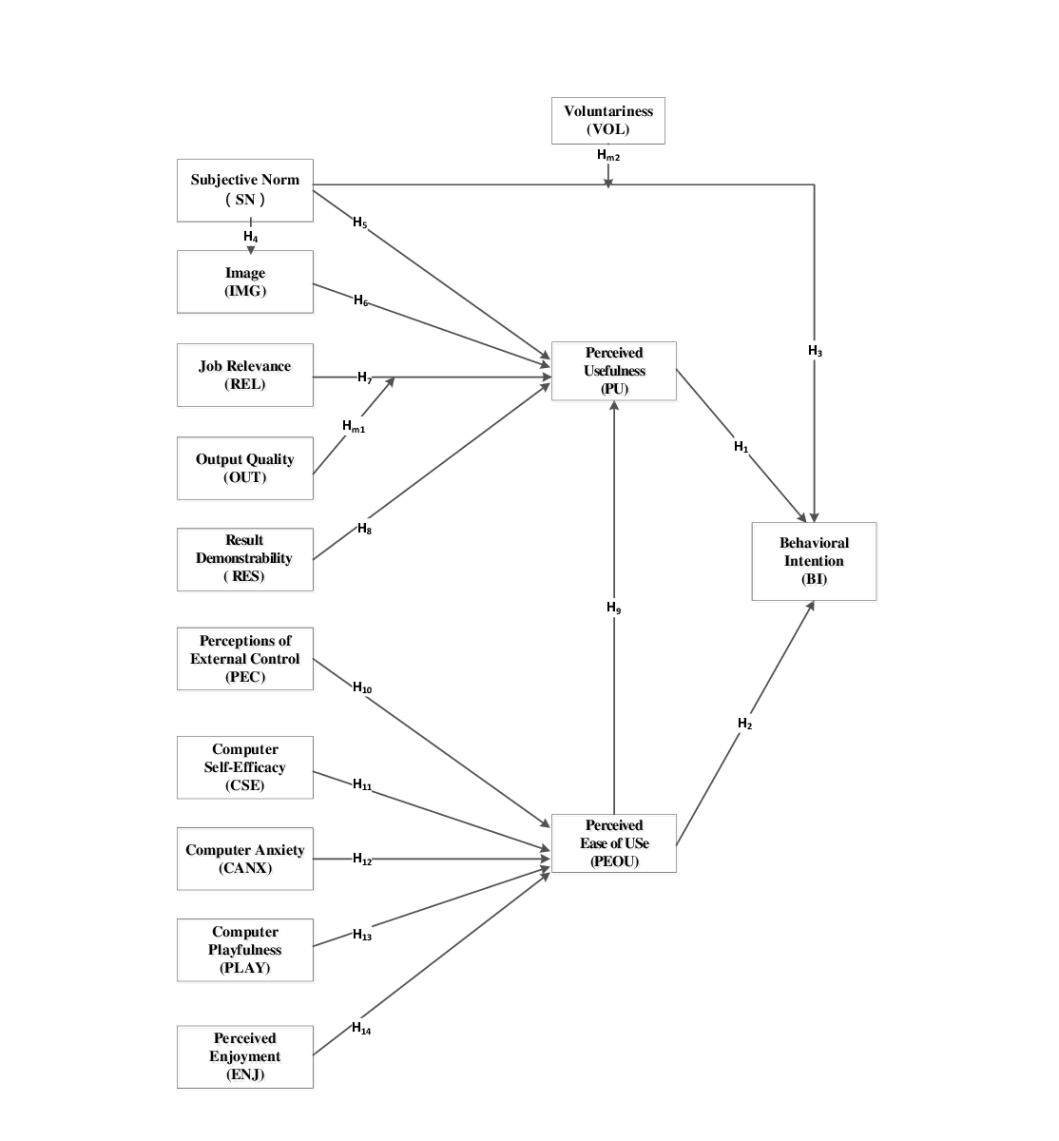
Modified technology acceptance model 3 adopted in this study. Hm1: hypothesis moderator 1; Hm2: hypothesis moderator 2.

This study proposed a modified version of TAM3, developed by Venkatesh and Bala [11], to express user acceptance of a care plan system. The study hypotheses are described as follows (Figure 1): (1) PU and PEOU have significant relationships with BI (H1 and H2, respectively); (2) the effects of SN on BI (H3), SN on IMG (H4), and SN and IMG on PU (H5 and H6, respectively) are related to social influence; (3) REL and RES represent the cognitive instrumental processes of PU (H7 and H8, respectively); (4) PEOU has a significant relationship with PU (H9); (5) the relationship of PEC, which refers to personnel beliefs, with PEOU (H10) is a facilitating condition; (6) the effects of CSE, PLAY, and CANX on PEOU (H11, H12, and H13, respectively) represent individual differences in terms of general beliefs about computers and computer use; and (7) ENJ can be adjusted to predict the PEOU of a system (H14). The degree to which the adjustment of objective usability determines the PEOU of a target system was not validated in this study.

Venkatesh and Bala [11] included output quality (OUT) as a moderating variable (Table 1). REL on PU was stronger when OUT was higher. In addition, to distinguish voluntary use from mandatory use, the researchers included voluntariness (VOL) as a moderator (Table 1) of the relationship between SN and BI.

### Study Design and Sample

This cross-sectional study was approved by the Medical Ethics Committee of the Tri-Service General Hospital (TSGHIRB No. B-104-13). The study period was from October 2015 to January 2016. All participants were registered nurses aged older than 20 years who had been using a care plan system for longer than 1 month. Using convenience sampling, 250 nurses were recruited from eight hospitals affiliated with public organizations in Taiwan. Data for this study were drawn from the same sample as that used in our previous study but were employed for different purposes and presented as a different type of data in this study. After informed consent was obtained from all participants, a structured questionnaire was employed for data collection.

Hair et al [19] proposed the estimation of the minimum sample size in partial least squares (PLS)–structural equation modeling (SEM) analysis with multiple regression models by applying Cohen [20] definitions; effect sizes of 0.02, 0.15, and 0.35 were considered small, medium, and large, respectively. To facilitate PLS-SEM analysis, the research sample size was calculated based on the recommendations of Hair et al [19]. The sample size was calculated using the G*Power 3.0 software program (UCLA) with a power of .80, a medium effect size of 0.15, and alpha set at .05 for multiple regression of the maximum number of variables in a construct in our research framework, with five predictors used. A minimum sample size of 92 was necessary. Furthermore, a minimum sample size of 200 is often recommended for PLS-SEM [21,22]. Therefore, considering the 25% attrition rate, we recruited 250 nurses. The valid questionnaires completed by 222 nurses were used for data analysis, yielding a response rate of 88.80%.

### Measures

Our questionnaire collected the demographic data of the nurses, and self-reported data were collected using the questionnaire about TAM3 designed by Venkatesh and Bala [11]. Following approval from the original author, 50 items in the modified TAM3 questionnaire composed the constructs investigated in our research model.

The modified TAM3 questionnaire consisted of the TAM constructs PU, PEOU, and BI; the determinants of PEOU (CSE, PEC, CANX, PLAY, and ENJ); the determinants of PU (SN, IMG, REL, and RES); and the moderators OUT and VOL. Except for the construct of CSE, items for all constructs were rated on a 7-point Likert scale ranging from 1 (strongly disagree) to 7 (strongly agree). The CSE items were measured on a 10-point Guttman scale ranging from 1 (strongly disagree) to 10 (strongly agree). The TAM3 questionnaire had high internal consistency reliability (Cronbach alpha ranging from .76 to .93) and high validity [11].

### Data Analysis

Descriptive statistics were employed using SPSS Statistics version 20.0 (IBM Corp) to analyze sociodemographic variables and use characteristics of the care plan system. We estimated the measurement model, tested the structural model, and analyzed the relationships among all variables through PLS-SEM in SmartPLS version 3.0 (University of Hamburg).

#### Measurement Model Estimation

In accordance with the model evaluation criteria proposed by Hair et al [19], we assessed reliability, convergent validity, and discriminant validity. Internal consistency reliability was ensured if the composite reliability (CR) scores of all constructs and Cronbach alpha were higher than .70. Indicator reliability was ensured if all indicators’ outer loadings were greater than .70. Convergent validity was confirmed if the average variance extracted (AVE) scores of all constructs were higher than .50. The square root of the AVE of each construct needed to be higher than the correlation between the latent variables, and all the indicators’ outer loadings on their own constructs had to be higher than their cross-loadings with other constructs to satisfy the requirements of discriminant validity.

#### Moderating Effect Estimation

The PLS approach in SmartPLS and moderated multiple regression analysis in SPSS version 20.0 for Windows were applied to analyze and interpret interactions. We used SmartPLS version 3.0 to analyze the coefficients of interaction terms. The significance of a moderator was confirmed by *t* value (*t* >1.96) for all interaction effects (path coefficients). SPSS version 20.0 for Windows was used to calculate the model fit (*R*^2^ without moderator), new model fit (*R*^2^ with moderator), difference between these *R*^2^ values, and significance of this difference for all endogenous latent variables.

#### Structural Model Analysis

To evaluate the multicollinearity of the structural model, two correlated variable correlation coefficients had to be <.85 [23]. A standardized root mean square residual (SRMR) lower than .10 indicated acceptable goodness of fit in the model [24]. The coefficient of determination values (*R*^2^) representing weak, moderate, and substantial were .25, .50, and .75, respectively [19]. PLS-SEM with a bootstrapping procedure was employed to test the study hypotheses and analyze the path coefficients (significance level=5%).

## Results

### Participant Characteristics

The respondents reported their sociodemographic characteristics and use of the target information system. Of the 222 nurses, 95.5% (212/222) were women and 4.5% (10/222) were men. In total, 4 (1.8%) had a senior vocational school degree in nursing, 88 (39.6%) had an associate degree, and 130 (58.6%) had a bachelor’s degree or higher. Most participants had more than 6 years of professional nursing experience (150/222, 67.6%). The use of health information technology for less than 6 years had the highest representation throughout the study sample (190/222, 85.6%). Most of the participants (141/222, 63.5%) did not feel under pressure when using a computer.

### Measurement Model Results

As presented in Multimedia Appendix 1, for internal consistency reliability, all Cronbach alpha scores for the study variables were higher than .70, and CR scores ranged from .84 to .96, which were all acceptable. The outer loadings of all indicators were above .70, which implied satisfactory indicator reliability (see Multimedia Appendix 1). Multimedia Appendix 1 indicates that AVE scores for all variables were above .64. This result satisfied the requirement for convergent validity. To confirm the discriminant validity of constructs, we examined whether the square root of the AVE from each construct (see Multimedia Appendix 1) exceeded the correlation between the constructs in the research model. Moreover, as presented in Multimedia Appendix 2, we ensured that all indicators had outer loadings in relation to their own latent variables that were higher than their cross-loadings with other constructs. Therefore, we concluded that the measurement model satisfied the criteria for internal consistency, indicator, convergent, and discriminant validity.

### Analysis of Moderating Effects

Moderated multiple regression analysis and PLS-SEM were employed to test the moderating effects. All test results are presented in Multimedia Appendix 3. The *t* values for all path coefficients were lower than 1.96, and differences among the *R*^2^ values of all endogenous latent variables were minor and nonsignificant. Therefore, VOL and OUT did not exert any moderating effects.

### Structural Model Analysis and Hypothesis Testing

**Figure 2:**
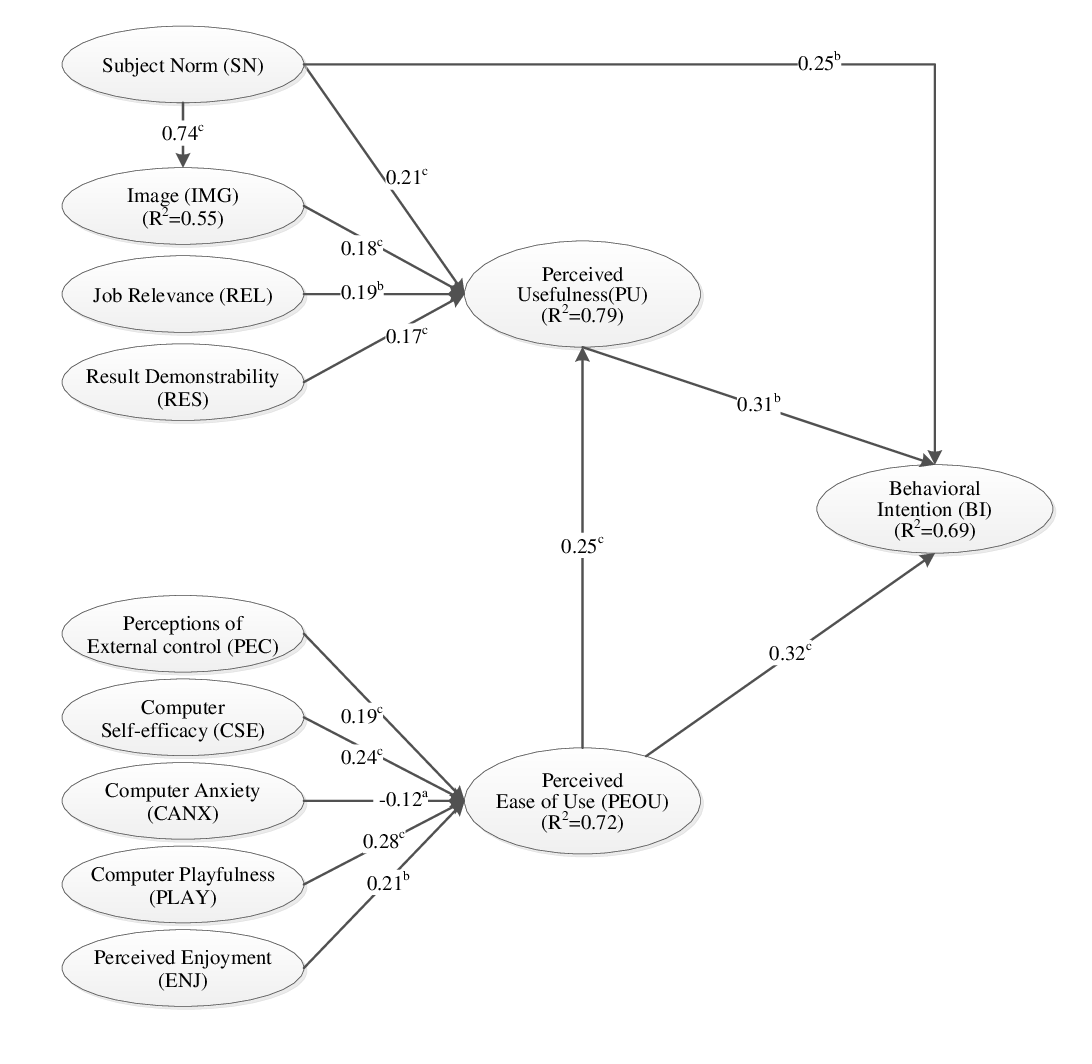
Analysis path of the structural model. ^a^*P*<.05. ^b^*P*<.01. ^c^*P*<.001. Note: No moderator variable was used in this model.

In this study, all bivariate correlations were lower than .85 (Multimedia Appendix 1). Therefore, multicollinearity was avoided. Multimedia Appendix 4 and Figure 2 present the explained variance of each construct. SN, IMG, REL, RES, and PEOU yielded approximately 79% of the variance for PU. The effects of CSE, PEC, CANX, PLAY, and ENJ on PEOU yielded approximately 72% of the total variance. The combination of SN, PEOU, and PU accounted for 69% of the variance observed for BI. This result indicated that the model explained high levels of variance.

In our research model, the SRMR was .09, which indicated good model fit of the data. Therefore, the model was considered acceptable. The total indirect effect and total effect of all constructs on BI toward using the care plan system are presented in Multimedia Appendix 4, including the total indirect effects of SN (.11) and PEOU (.08) on BI. Combined with the direct effect, the total effects of SN (.36), PU (.31), and PEOU (.40) on BI were calculated.

A bootstrapping procedure was used to calculate the statistical significance of all path coefficients. Our researchers selected 5000 samples and recruited 222 nurses to estimate the path coefficients. As indicated by the PLS analysis results presented in Figure 2 and Multimedia Appendix 3 and Multimedia Appendix 4, all study hypotheses were supported by the data. The results revealed that SN, PEOU, and PU (path coefficients range=.25–.32) were all significant determinants of BI (*R*^2^=.69). SN, IMG, REL, RES, and PEOU (path coefficients range=.17–.25) all had a significant effect on PU (*R*^2^=.79). PEOU (*R*^2^=.72) was significantly influenced by PEC, CSE, PLAY, ENJ (path coefficients range=.19–.28) and CANX (path coefficient=−.12).

## Discussion

### Principal Findings

This study is the first to reveal the ability of TAM3 to comprehensively explore the determinants of BI for use of a care plan system. Our results indicated that the research model accounted for 69% of the variance in the care plan system, and all hypotheses supported the use of TAM3 except for the nonsignificant moderating effects of VOL and OUT. Few studies in nursing settings have explored user acceptance based on the determinants of PEOU and the combination of such determinants with those of PU. Our study empirically demonstrated that the determinants of PEOU influence PEOU and the determinants of PU and PEOU influence PU and SN, PEOU, with PU consequently predicting BI. Well-organized health information technology positively influences nurses’ intentions to use a care plan system in professional settings [25]. This study provided an innovative methodology for evaluating and understanding nurses’ acceptance of and need for a care plan system to implement well-organized health information technology and improve performance in nursing practice.

Using the modified TAM3, our research model explained 69% of the total variance, which was more than that explained by other TAM studies [12-15]. Our study results demonstrated that TAM3 is highly suitable for determining nurses’ perceptions of using health information technology in nursing settings. Wu and Shen [26] indicated that PEOU, PU, and SN all had direct effects on health care professionals’ BI. Moreover, in health care environments, PEOU and PU are key factors influencing the acceptance of health information technology by nursing personnel [12,15,27]. Using TAM3 with PU, PEOU, and SN to analyze users’ BIs, we observed 69% variance for BI. In addition, SN, PEOU, and PU all had strong positive effects on BI, with path coefficients of .25 (*P*<.001), .32 (*P*<.001), and .31 (*P*<.01), respectively. In this study, the significant total effects of SN (path coefficient=.36), PU (path coefficient=.31), and PEOU (path coefficient=.40) on BI were also notable. Therefore, we assumed that the constructs of SN, PEOU, and PU are powerful predictors of nurses’ BI to use a care plan system and contribute to the substantial explained variance of the modified TAM3. We suggest that implementing new health information technology in routine nursing care would improve related performance in nursing practice [25], broaden professional perspectives, and highlight preferences to enhance the ease of use of health information technology and improve key individual’s opinions regarding the use of health information technology.

Using the modified TAM3, this study empirically verified the collected data and confirmed that the determinants of PEOU for measuring nurses’ BI as well as all the determinants of PEOU had significant relationships with PEOU and explained 72% of the variance of PEOU. Moreover, we observed that PEOU had not only the most significant influence on BI to use the care plan system but also the strongest direct effect on BI. This result differed from those of previous studies [12,15,25]. In the pooled data for TAM3, PEC, CSE, PLAY (path coefficients range=.15–.33), and CANX (path coefficient=−.18) had a direct relationship with PEOU, and the total explained variance for PEOU was 52% [11]. The result of our research proved that PEC, CSE, PLAY, ENJ (path coefficients range=.19–.28), and CANX (path coefficient=−.12) significantly influence PEOU and jointly explain 72% of the variance in PEOU (*R*^2^=.72). We posit that this research model with all the determinants of PEOU differed considerably from those used in previous studies, which adopted the modified TAM or the determinants of PU. That is, the TAM3 model provides a comprehensive set of PEOU determinants and an exhaustive explanation of the power of the PEOU of a care plan system. On the basis of our results, we recommend increasing individuals’ BI to use computers to perform speciﬁc tasks, increase cognitive spontaneity related to computers, enhance enjoyment during the use of a target health information technology system, and reduce the level of fear in individuals’ interactions with health information technology to promote nurses’ PEOU toward the care plan system.

By comparing the direct effect of SN, IMG, REL, RES, and PEOU on PU in this study with the pooled data for TAM3 [11], we obtained SN, IMG, REL, RES, and PEOU values of .21/.04, .18/.24, .19/.03, .17/.26, and .25/.08, respectively. In this study, the determinants of PU explained 79% of the variance in PU (*R*^2^=.79). As indicated in the pooled data of TAM3, PU is jointly predicted by the determinants of PU, with 67% of the total variance explained (*R*^2^=.67) [11]. By contrast, other studies that adopted the modified model with the determinants of PU have predicted that PU accounts for 46% to 59% of the explained variance [15,28]. Our results were consistent with those of some previous studies [15,28], where nurses’ PU of a care plan system was enhanced when they perceived that key individuals wanted them to use the health information technology in question. In addition, nurses’ social status is enhanced when using said health information technology. Moreover, the significant relationships of REL (path coefficient=.19) and RES (path coefficient=.17) with PU in our study indicated that the nurses perceived health information technology as appropriate to their work. More tangibly, health information technology had a positive influence on the PU of the care plan system. When a user perceives that health information technology is useful, they also believe that it is easy to use [15]. Another key finding in our study was that PEOU had the most significant effect on PU. The determinants of PU in TAM3 are appropriate constructs for evaluating user belief regarding the usefulness of health information technology in nursing settings.

Venkatesh and Bala [11] argued that VOL and OUT are influential moderating variables in contexts where information technology is used. By contrast, our results revealed that the moderating variables VOL and OUT had no significant effects on the care plan system. Sun and Zhang [29] indicated that a weaker moderating effect elicits a stronger response from a more experienced user. The moderating effect of VOL weakens over time. Zhang and Cocosila [15] reported that the experience moderator did not influence homecare nurses’ beliefs regarding the use of information technology. We assumed that all of our participants had accumulated considerable experience of using a care plan system and that this led to the aforementioned nonsignificant moderating effects. The other reason for these effects may have been that our study adopted cross-sectional quantitative data to determine user acceptance, whereas TAM3 has generally been employed in longitudinal ﬁeld studies. Therefore, the moderators had no significant effects.

### Limitations and Recommendations

The first limitation of this study is that the cross-sectional data used were all collected at the same time. This could have yielded nonsignificant moderating effects. Moreover, our participants had already used the care plan system for more than 1 month. Therefore, this may have led to the moderating effects of VOL on the bivariables weakening with increasing experience. To avoid confusion in the results, the experience moderator was not measured in this study. We recommend that in the future, researchers explore the factors of user acceptance in the early stages of health information technology implementation and conduct longitudinal ﬁeld studies.

Second, individual knowledge, attitude, and skill level with respect to nursing are crucial for designing patient-centered care plans and improving patient care quality [2,30]. Because the decision-making aspect of care planning varies from person to person and nursing students have insufficient nursing knowledge to design suitable care plans for patients, the measurement of objective usability—a comparison between the amounts of time spent by an expert and a novice to perform a task using the system—is conflicted. Therefore, we did not examine the objective usability variable in this study. To examine the relationship between objective usability and PEOU, future studies could employ a simple operating system, such as a patient physical data record system.

In health care, information technology developments adapt to changing needs [14]. To increase the use of information technology and improve its performance, we recommend that health care institutions adopt a model that measures nurses’ perceptions of health information technology use to identify why the implementation of health information technology is accepted or rejected.

### Conclusion

We applied TAM3 [11] to validate and measure determinants that affect the BI of nurses to use a care plan system. The critical determinants affecting nurses’ acceptance of a care plan system were empirically examined. The results emphasize that SN, PEOU, and PU all predicted users’ BI to use the care plan system, and the determinants of PU and PEOU significantly influenced PU and PEOU. This research contributes to the exploration of user acceptance and to a better understanding of care plan system use in routine nursing practice.

## Appendix

Multimedia Appendix 1 Cronbach alpha, composite reliability, outer loadings, average variance extracted, and Pearson correlation coefficients for the construct variables.

Multimedia Appendix 2 Outer loadings and cross-loadings of the study variables.

Multimedia Appendix 3 Path coefficients and results of the moderating effects analysis and research hypotheses.

Multimedia Appendix 4 Results of hypothesis testing, R2 calculation, and determining the total effect and total indirect effect for all variables with respect to behavioral intention to use.

## Abbreviations

AVE: average variance extracted
BI: behavioral intention
CANX: computer anxiety
CR: composite reliability
CSE: computer self-efficacy
ENJ: perceived enjoyment
IMG: image
OUT: output quality
PEC: perception of external control
PEOU: perceived ease of use
PLAY: computer playfulness
PLS: partial least squares
PU: perceived usefulness
REL: job relevance
RES: result demonstrability
SEM: structural equation modeling
SN: subjective norm
SRMR: standardized root mean square residual
TAM: technology acceptance model
VOL: voluntariness
