# Supplementary material for: Determining Factors Affecting Nurses’ Acceptance of a Care Plan System Using a Modified Technology Acceptance Model 3: Structural Equation Model With Cross-Sectional Data
Source: JMIR Med Inform. 2020 May 5;8(5):e15686. doi: 10.2196/15686 (PMC7238093; doi:10.2196/15686)
Supplement: Multimedia Appendix 1 [file medinform_v8i5e15686_app1.docx]

Multimedia Appendix 1. Cronbach alpha, CR, outer loadings, AVE, and Pearson’s correlation coefficients for the construct variables.

| Variable | Cronbach’s α | CR | Outer loading | AVE | VOL | SN | IMG | REL | OUT | RES | PEC | CSE | CANX | PLAY | ENJ | PEOU | PU | BI |
| --- | --- | --- | --- | --- | --- | --- | --- | --- | --- | --- | --- | --- | --- | --- | --- | --- | --- | --- |
| VOL | .80 | .86 | 0.70–0.93 | .68 | **.82^c^** |  |  |  |  |  |  |  |  |  |  |  |  |  |
| SN | .80 | .88 | 0.82–0.85 | .71 | .52 | **.84^c^** |  |  |  |  |  |  |  |  |  |  |  |  |
| IMG | .79 | .87 | 0.80–0.87 | .69 | .54 | .74 | **.83^c^** |  |  |  |  |  |  |  |  |  |  |  |
| REL | .83 | .89 | 0.85–0.87 | .73 | .58 | .77 | .70 | **.86^c^** |  |  |  |  |  |  |  |  |  |  |
| OUT | .90 | .94 | 0.90–0.92 | .84 | .57 | .76 | .72 | .77 | **.91^c^** |  |  |  |  |  |  |  |  |  |
| RES | .82 | .89 | 0.80–0.90 | .73 | .43 | .63 | .55 | .66 | .65 | **.86^c^** |  |  |  |  |  |  |  |  |
| PEC | .90 | .95 | 0.95–0.96 | .91 | .51 | .60 | .58 | .76 | .72 | .58 | **.95^c^** |  |  |  |  |  |  |  |
| CSE | .79 | .87 | 0.71–0.90 | .70 | .50 | .65 | .61 | .64 | .67 | .54 | .57 | **.84^c^** |  |  |  |  |  |  |
| CANX | .74 | .84 | 0.73–0.84 | .64 | −.35 | −.42 | −.34 | −.38 | −.39 | −.48 | −.34 | −.28 | **.80^c^** |  |  |  |  |  |
| PLAY | .86 | .90 | 0.72–0.89 | .71 | .55 | .63 | .68 | .69 | .68 | .52 | .59 | .61 | −.50 | **.84^c^** |  |  |  |  |
| ENJ | .86 | .91 | 0.86–0.91 | .78 | .48 | .65 | .62 | .61 | .69 | .56 | .62 | .73 | −.50 | .73 | **.88^c^** |  |  |  |
| PEOU | .89 | .92 | 0.82–0.90 | .75 | .53 | .72 | .69 | .78 | .76 | .68 | .73 | .65 | −.50 | .75 | .73 | **.87^c^** |  |  |
| PU | .94 | .96 | 0.94–0.95 | .90 | .52 | .78 | .74 | .79 | .79 | .70 | .60 | .59 | −.40 | .63 | .66 | .80 | **.95^c^** |  |
| BI | .85 | .91 | 0.82–0.92 | .77 | .52 | .73 | .66 | .76 | .75 | .60 | .60 | .63 | −.51 | .71 | .71 | .76 | .77 | **.88^c^** |

^a^CR: Composite reliability.

^b^AVE: Average variance extracted.

^c^The square root of the AVE for each latent variable.

^d^VOL: Voluntariness

^e^SN: Subjective norm

^f^IMG: Image

^g^REL: Job relevance

^h^OUT: Output quality

^i^RES: Result demonstrability

^j^PEC: Perception of external control

^k^CSE: Computer self-efficacy

^l^CANX: Computer anxiety

^m^PLAY: Computer playfulness

^n^ENJ: Perceived enjoyment

^o^PEOU: Perceived ease of use

^p^PU: Perceived usefulness

^q^BI: Behavioral intention
