# Supplementary material for: Determining Factors Affecting Nurses’ Acceptance of a Care Plan System Using a Modified Technology Acceptance Model 3: Structural Equation Model With Cross-Sectional Data
Source: JMIR Med Inform. 2020 May 5;8(5):e15686. doi: 10.2196/15686 (PMC7238093; doi:10.2196/15686)
Supplement: Multimedia Appendix 2 [file medinform_v8i5e15686_app2.docx]

Multimedia Appendix 2. Outer loadings and cross-loadings of the study variables.

| Construct | Label | Component | | | | | | | | | | | | | | |
| --- | --- | --- | --- | --- | --- | --- | --- | --- | --- | --- | --- | --- | --- | --- | --- | --- |
|  |  | VOL | SN | IMG | REL | OUT | RES | PEC | CSE | CANX | PLAY | ENJ | PEOU | PU | BI |  |
| VOL | VOL1 | **.93** | .54 | .57 | .57 | .56 | .43 | .55 | .49 | −.44 | .61 | .54 | .56 | .51 | .59 |  |
|  | VOL2 | **.70** | .22 | .35 | .28 | .33 | .16 | .24 | .33 | −.02 | .33 | .20 | .28 | .26 | .21 |  |
| SN | SN1 | .36 | **.85** | .55 | .57 | .52 | .46 | .50 | .46 | −.36 | .46 | .50 | .50 | .48 | .59 |  |
|  | SN2 | .44 | **.85** | .62 | .57 | .56 | .46 | .54 | .54 | −.31 | .50 | .49 | .51 | .49 | .55 |  |
|  | SN3 | .42 | **.82** | .68 | .75 | .77 | .62 | .57 | .52 | −.37 | .60 | .61 | .74 | .91 | .68 |  |
| IMG | IMG1 | .50 | .70 | **.83** | .72 | .75 | .62 | .60 | .54 | −.40 | .63 | .58 | .74 | .82 | .68 |  |
|  | IMG2 | .46 | .62 | **.87** | .51 | .52 | .39 | .49 | .46 | −.25 | .58 | .53 | .48 | .51 | .50 |  |
|  | IMG3 | .44 | .49 | **.80** | .48 | .47 | .28 | .37 | .43 | −.14 | .46 | .42 | .41 | .43 | .40 |  |
| REL | REL1 | .40 | .60 | .49 | **.86** | .58 | .47 | .49 | .67 | −.27 | .55 | .46 | .60 | .53 | .59 |  |
|  | REL2 | .48 | .74 | .72 | **.87** | .77 | .68 | .61 | .62 | −.41 | .62 | .60 | .78 | .88 | .71 |  |
|  | REL3 | .47 | .58 | .53 | **.85** | .57 | .47 | .50 | .70 | −.26 | .58 | .46 | .56 | .51 | .61 |  |
| OUT | OUTQ1 | .53 | .63 | .64 | .70 | **.90** | .58 | .60 | .74 | −.33 | .61 | .61 | .67 | .69 | .65 |  |
|  | OUTQ2 | .48 | .75 | .67 | .71 | **.92** | .60 | .61 | .60 | −.37 | .63 | .64 | .70 | .77 | .72 |  |
|  | OUTQ3 | .50 | .69 | .68 | .71 | **.92** | .60 | .61 | .65 | −.36 | .62 | .63 | .72 | .72 | .69 |  |
| RES | RES1 | .28 | .49 | .50 | .49 | .48 | **.80** | .39 | .45 | −.33 | .38 | .44 | .51 | .55 | .46 |  |
|  | RES2 | .41 | .53 | .50 | .58 | .63 | **.90** | .49 | .54 | −.45 | .52 | .51 | .61 | .63 | .55 |  |
|  | RES3 | .27 | .60 | .43 | .62 | .57 | **.86** | .51 | .51 | −.45 | .43 | .50 | .61 | .62 | .52 |  |
| PEC | PEC1 | .47 | .56 | .54 | .75 | .67 | .53 | **.95** | .55 | −.25 | .55 | .52 | .60 | .54 | .59 |  |
|  | PEC2 | .48 | .59 | .57 | .70 | .71 | .58 | **.96** | .54 | −.28 | .60 | .56 | .64 | .59 | .61 |  |
| CSE | CSE1 | .41 | .61 | .55 | .61 | .59 | .53 | .50 | **.88** | −.34 | .55 | .54 | .69 | .60 | .58 |  |
|  | CSE2 | .46 | .55 | .55 | .54 | .59 | .44 | .53 | **.90** | −.27 | .53 | .48 | .56 | .45 | .51 |  |
|  | CSE4 | .43 | .44 | .40 | .42 | .49 | .36 | .41 | **.71** | −.22 | .39 | .52 | .42 | .44 | .40 |  |
| CANX | ANX1 | −.44 | −.49 | −.48 | −.44 | −.46 | −.45 | −.40 | −.32 | **.84** | −.54 | −.56 | −.53 | −.45 | −.56 |  |
|  | ANX2 | −.11 | −.13 | −.04 | −.15 | −.14 | −.27 | −.13 | −.12 | **.73** | −.22 | −.21 | −.23 | −.18 | −.26 |  |
|  | ANX3 | −.16 | −.23 | −.13 | −.21 | −.20 | −.37 | −.18 | −.14 | **.82** | −.32 | −.30 | −.33 | −.23 | −.28 |  |
| PLAY | PLAY1 | .55 | .60 | .59 | .65 | .63 | .51 | .57 | .52 | −.52 | **.88** | .75 | .72 | .62 | .69 |  |
|  | PLAY2 | .46 | .56 | .63 | .58 | .60 | .40 | .50 | .55 | −.35 | **.86** | .58 | .59 | .53 | .62 |  |
|  | PLAY3 | .55 | .52 | .62 | .60 | .59 | .39 | .48 | .54 | −.35 | **.89** | .57 | .62 | .52 | .59 |  |
|  | PLAY4 | .42 | .42 | .44 | .46 | .45 | .42 | .42 | .43 | −.44 | **.72** | .55 | .58 | .44 | .48 |  |
| ENJ | ENJ1 | .44 | .56 | .51 | .51 | .53 | .41 | .50 | .41 | −.45 | .63 | **.87** | .62 | .54 | .61 |  |
|  | ENJ2 | .40 | .58 | .58 | .56 | .66 | .53 | .55 | .60 | −.37 | .59 | **.86** | .63 | .62 | .60 |  |
|  | ENJ3 | .45 | .58 | .56 | .55 | .63 | .55 | .56 | .50 | −.51 | .72 | **.91** | .68 | .58 | .68 |  |
| PEOU | PEOU1 | .47 | .69 | .66 | .72 | .67 | .61 | .66 | .60 | −.36 | .65 | .63 | **.87** | .75 | .67 |  |
|  | PEOU2 | .46 | .64 | .61 | .70 | .68 | .63 | .60 | .58 | −.44 | .63 | .63 | **.88** | .71 | .65 |  |
|  | PEOU3 | .51 | .59 | .57 | .66 | .66 | .58 | .57 | .54 | −.52 | .65 | .66 | **.90** | .70 | .66 |  |
|  | PEOU4 | .38 | .58 | .53 | .64 | .63 | .53 | .55 | .55 | −.43 | .68 | .62 | **.82** | .60 | .65 |  |
| PU | PU1 | .47 | .71 | .71 | .78 | .79 | .65 | .57 | .55 | −.40 | .62 | .65 | .77 | **.94** | .73 |  |
|  | PU2 | .44 | .69 | .71 | .73 | .72 | .68 | .58 | .55 | −.36 | .57 | .58 | .73 | **.94** | .71 |  |
|  | PU3 | .47 | .73 | .69 | .75 | .74 | .67 | .56 | .59 | −.39 | .60 | .63 | .76 | **.95** | .76 |  |
| BI | BI1 | .46 | .58 | .50 | .63 | .60 | .52 | .48 | .50 | −.50 | .60 | .63 | .66 | .67 | **.90** |  |
|  | BI2 | .43 | .63 | .55 | .67 | .63 | .54 | .56 | .52 | −.47 | .62 | .60 | .70 | .71 | **.92** |  |
|  | BI3 | .51 | .73 | .69 | .70 | .75 | .52 | .55 | .63 | −.37 | .66 | .64 | .64 | .66 | **.82** |  |

^a^VOL: Voluntariness

^b^SN: Subjective norm

^c^IMG: Image

^d^REL: Job relevance

^e^OUT: Output quality

^f^RES: Result demonstrability

^g^PEC: Perception of external control

^h^CSE: Computer self-efficacy

^i^CANX: Computer anxiety

^j^PLAY: Computer playfulness

^k^ENJ: Perceived enjoyment

^l^PEOU: Perceived ease of use

^m^PU: Perceived usefulness

^n^BI: Behavioral intention
