# Supplementary material for: Determining Factors Affecting Nurses’ Acceptance of a Care Plan System Using a Modified Technology Acceptance Model 3: Structural Equation Model With Cross-Sectional Data
Source: JMIR Med Inform. 2020 May 5;8(5):e15686. doi: 10.2196/15686 (PMC7238093; doi:10.2196/15686)
Supplement: Multimedia Appendix 3 [file medinform_v8i5e15686_app3.docx]

Multimedia Appendix 3. Path coefficients and results of the moderating effects analysis and research hypotheses.

| Hypothesis | Dependent variable | Independent variable | moderator | Path coefficient | | Model fit | | | Result |
| --- | --- | --- | --- | --- | --- | --- | --- | --- | --- |
|  |  |  |  | Path coefficient | *t* value | R^2^  without moderator | R^2^  with moderator | R^2^  difference |  |
| H_1_ | Perceived usefulness | Behavioral intention |  | .31 | 3.07 |  |  |  | Supported |
| H_2_ | Perceived ease of use | Behavioral intention |  | .32 | 4.11 |  |  |  | Supported |
| H_3_ | Subjective norm | Behavioral intention |  | .25 | 3.22 |  |  |  | Supported |
| H_4_ | Subjective norm | Image |  | .74 | 22.39 |  |  |  | Supported |
| H_5_ | Subjective norm | Perceived usefulness |  | .21 | 3.61 |  |  |  | Supported |
| H_6_ | Image | Perceived usefulness |  | .18 | 3.52 |  |  |  | Supported |
| H_7_ | Job relevance | Perceived usefulness |  | .19 | 3.21 |  |  |  | Supported |
| H_8_ | Result demonstrability | Perceived usefulness |  | .17 | 3.48 |  |  |  | Supported |
| H_9_ | Perceived ease of use | Perceived usefulness |  | .25 | 3.78 |  |  |  | Supported |
| H_10_ | Perception of external control | Perceived ease of use |  | .19 | 3.47 |  |  |  | Supported |
| H_11_ | Computer self-efficacy | Perceived ease of use |  | .24 | 3.88 |  |  |  | Supported |
| H_12_ | Computer anxiety | Perceived ease of use |  | −.12 | 2.57 |  |  |  | Supported |
| H_13_ | Computer playfulness | Perceived ease of use |  | .28 | 4.16 |  |  |  | Supported |
| H_14_ | Perceived enjoyment | Perceived ease of use |  | .21 | 2.95 |  |  |  | Supported |
| H_m1_ | Job relevance | Perceived usefulness | Output quality | −.004 | .12 | .684 | .685 | .001 | Not supported |
| H_m2_ | Subjective norm | Behavioral intention | Voluntariness | −.021 | .56 | .547 | .549 | .002 | Not supported |
