# Supplementary material for: Determining Factors Affecting Nurses’ Acceptance of a Care Plan System Using a Modified Technology Acceptance Model 3: Structural Equation Model With Cross-Sectional Data
Source: JMIR Med Inform. 2020 May 5;8(5):e15686. doi: 10.2196/15686 (PMC7238093; doi:10.2196/15686)
Supplement: Multimedia Appendix 4 [file medinform_v8i5e15686_app4.docx]

Multimedia Appendix 4. Results of hypothesis testing, R^2^ calculation, and determining the total effect and total indirect effect for all variables with respect to behavioral intention to use.

| Variable | R^2^ | Direct effect | | | | Total indirect effect | | Total effect | | | |
| --- | --- | --- | --- | --- | --- | --- | --- | --- | --- | --- | --- |
|  |  | IMG | PEOU | PU | BI | PU | BI | IMG | PEOU | PU | BI |
| Subjective norm |  | .74^c^ |  | .21^c^ | .25^c^ | .14^c^ | .11^b^ | .74^c^ |  | .35^c^ | .36^c^ |
| Image | .55 |  |  | .18^c^ |  |  | .06^a^ |  |  | .18^c^ | .06^a^ |
| Job relevance |  |  |  | .19^c^ |  |  | .06 |  |  | .19^c^ | .06 |
| Result demonstrability |  |  |  | .17^c^ |  |  | .06^a^ |  |  | .17^c^ | .06^a^ |
| Perception of external control |  |  | .19^b^ |  |  | .05^b^ | .08^b^ |  | .19^b^ | .05^b^ | .08^b^ |
| Computer self-efficacy |  |  | .24^c^ |  |  | .06^b^ | .10^c^ |  | .24^c^ | .06^b^ | .10^c^ |
| Computer anxiety |  |  | −.12^b^ |  |  | −.03^a^ | −.05^a^ |  | −.12^b^ | −.03^a^ | −.05^a^ |
| Computer playfulness |  |  | .28^c^ |  |  | .07^b^ | .11^c^ |  | .28^c^ | .07^b^ | .11^c^ |
| Perceived enjoyment |  |  | .21^b^ |  |  | .05^a^ | .08^a^ |  | .21^b^ | .05^a^ | .08^a^ |
| Perceived ease of use | .72 |  |  | .25^c^ | .32^c^ |  | .08^a^ |  |  | .25^c^ | .40^c^ |
| Perceived usefulness | .79 |  |  |  | .31^b^ |  |  |  |  |  | .31^b^ |
| Behavioral intention | .69 |  |  |  |  |  |  |  |  |  |  |

^a^*P*<.05.

^b^*P*<.01.

^c^*P*<.001.

^d^IMG: Image

^e^PEOU: Perceived ease of use

^f^PU: Perceived usefulness

^g^BI: Behavioral intention
